# Lipid and Bile Acid Dysmetabolism in Crohn's Disease

**DOI:** 10.1155/2018/7270486

**Published:** 2018-10-01

**Authors:** Koji Uchiyama, Hisashi Kishi, Wataru Komatsu, Masanori Nagao, Shuji Ohhira, Gen Kobashi

**Affiliations:** ^1^Laboratory of International Environmental Health, Center for International Cooperation, Dokkyo Medical University, Tochigi 321-0293, Japan; ^2^Department of Public Health, Dokkyo Medical University School of Medicine, Tochigi 321-0293, Japan

## Abstract

Crohn's disease is one of the systemic autoimmune diseases. It commonly affects the small intestine and colon but may involve any portion of the gastrointestinal tract from the mouth to the anus. The most affected area by Crohn's disease is the distal part of the small intestine, in which the bile acid molecules are most efficiently reabsorbed. Bile acids form mixed micelles together with fatty acids, which function as a transport vehicle to deliver fatty acids to the apical membrane of enterocytes for absorption. Therefore, if the terminal ileum is impaired, bile acid malabsorption may occur, which may cause congenital diarrhoea in Crohn's disease. Similarly, the impairment of the terminal ileum also induces fatty acid malabsorption, which may influence the role of fatty acids in Crohn's disease. In contrast, a recent study reported that multidrug resistance protein 1 (MDR1) regulated effector T-cell function in the ileum from bile acid-driven oxidative stress and MDR1 loss of function in a subset of patients with Crohn's disease. However, the role of consumption of fatty acids in Crohn's disease remains to be fully elucidated. This review is aimed at providing an overview of some recent developments in research of Crohn's disease from comprehensive perspective with a focus on the connection between disease location and behaviour, lipid diets, and bile acid malabsorption.

## 1. Introduction

Crohn's disease is one of the main disease phenotypes of inflammatory bowel disease. It is often characterised by systemic symptoms and discontinuous lesions with inflammation that can involve the full thickness of the affected portion of the bowel from mucosa to serosa. The small intestine and the colon are the most affected areas, but any portion of the gastrointestinal tract from the mouth to the anus can be involved.

Crohn's disease is considered to be a multifactorial disease with both genetic and acquired factors in its aetiology. Genetic studies highlighted the importance of the intestinal immune system, including dysregulation of intestinal CD4^+^ T-cell subgroups [1, 2]. Meanwhile, as discussed in the European Crohn's and Colitis Organisation's Epidemiological Committee study [3], the “westernised” lifestyle, such as an increased consumption of refined sugar, fatty acids, and fast food and a reduced consumption of fruits, vegetables, and fibres, was deemed to link to the development of Crohn's disease. In parallel, the first choice of therapy in children with Crohn's disease is exclusive enteral nutrition, which is an induction therapy [4]. Enteral nutrition is one of the established remission–induction therapies for both children and adults with Crohn's disease in Japanese population [5]. However, it is not a common treatment in adults with Crohn's disease in western countries [6], because no studies on its significant efficacy were found in systematic reviews [7]. Interestingly, a recent review showed an evidence to support a possible role of exclusive enteral nutrition in a newly diagnosed adult with Crohn's disease with ileal involvement [6].

This review is aimed at providing an overview of some recent developments in research concerning the molecular pathways of lipid consumptions, which may connect immunological and nutritional studies in Crohn's disease and epidemiological studies on disease location, phenotype, and diet.

## 2. Location and Phenotype of Crohn's Disease

**Figure 1:**
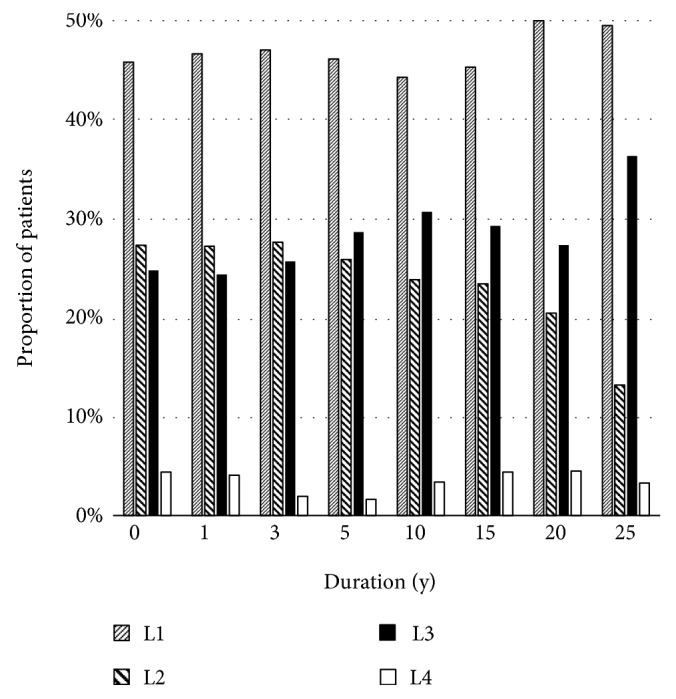
Disease location at diagnosis and at elapsed years among patients with Crohn's disease in a study of the University Hospital of Liège in Belgium [11].

**Figure 2:**
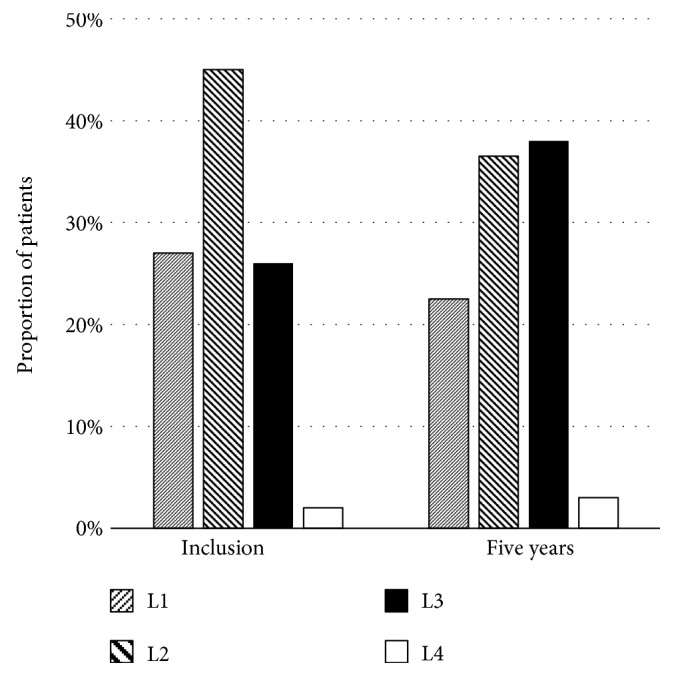
Disease location at recruitment and after 5 years among patients with Crohn's disease in a study in southeastern Norway [12].

**Figure 3:**
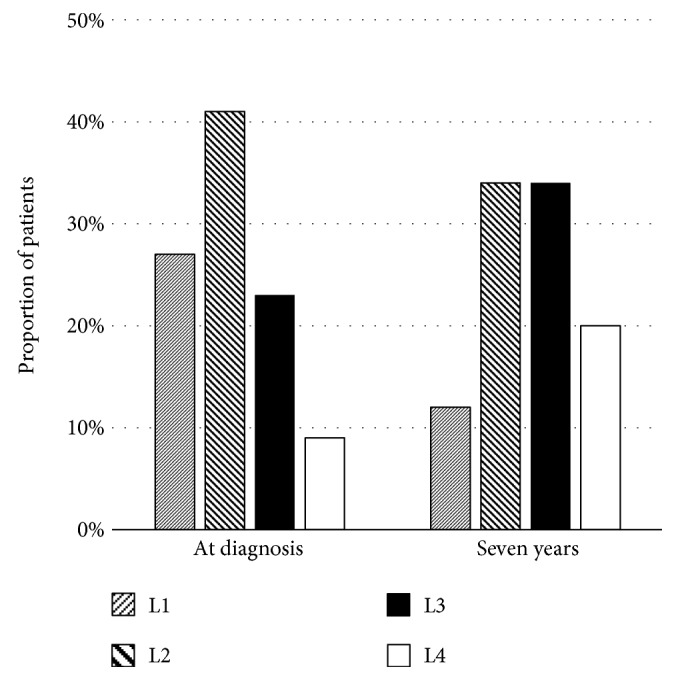
Disease location at diagnosis and after 7 years among patients with Crohn's disease in a study of a Danish cohort [10].

Generally, the terminal ileum and colon are the commonest locations affected in Crohn's disease, of which approximately 50% of patients have involvement [8, 9]. About 30% of the patients have only small bowel involvement, and the remaining 20% of the patients have isolated colonic involvement [8]. Changes in disease location are likely to occur over time in about 5–24% of patients with the disease [10–12]. In the Montreal classification, the disease location was defined as follows: L1, terminal ileum; L2, colon; L3, ileocolon; and L4, upper gastrointestinal tract [13]. Figures 1–3 show three studies, which reported disease location changes [10–12]. Their results suggest that only ileal or colonic involvement tends to occur at disease onset. However, as time progresses, the disease location extends to the other organs; especially both the ileum and colon (L3) will be involved. In the study of a Danish cohort [10], biologics, infliximab or adalimumab, showed protective effects on changes in disease location. Infliximab and adalimumab are anti-tumour necrosis factor-*α* (TNF-*α*) antibody drugs. The result of the above study may suggest that inflammatory cytokines, such as TNF-*α*, play an important role in the expansion of the disease location.

The phenotype of Crohn's disease was defined as B1, nonstricturing and nonpenetrating (inflammation); B2, stricturing; and B3, penetrating in the Montreal classification [13]. At diagnosis, the predominance of inflammatory behaviour (B1) is the most prevalent phenotype in patients with Crohn's disease [14]. However, dramatic changes in the proportion of disease behaviour were reported [14–16]. Usually, inflammation (B1) evolves to stricturing (B2) or penetrating (B3). This suggests that inflammation influences factors that may cause the exacerbation of Crohn's disease.

## 3. Epidemiology of Diet and Crohn's Disease

Several large longitudinal studies have reported dietary information prior to the onset of Crohn's disease. An association was found between long-term intakes of dietary fibre [17] and fish [18] and high intake of zinc [19] and lower risk of Crohn's disease in the Nurses' Health Study (NHS). Several systematic reviews also reported dietary factors that were related to the development of Crohn's disease. A late meta-analysis of 12 studies revealed that 570 patients with Crohn's disease had a significantly higher odds ratio of vitamin D deficiency than 778 controls [20]. A recent systematic review of 19 studies reported that high intakes of total fats, total polyunsaturated fatty acids (PUFAs), n-6 PUFAs, and meat were associated with an increased risk of Crohn's disease [21].

Several potential mechanisms, which could explain an inverse association with fibre, zinc, and vitamin D, were proposed. Short-chain fatty acids, mainly acetate, propionate, and butyrate, are produced in the large bowel by anaerobic bacterial fermentation of fibre [22, 23]. Butyrate was reported to decrease inflammatory cytokine expression, such as TNF-*α*, via inhibition of nuclear factor-kappa B (NF-*κ*B) activation [22, 24]. TNF-*α* has an effect on activation of T helper-1 (Th1) immune responses [24, 25]. Therefore, fibre consumption may regulate Th1 immune responses in Crohn's disease. In a recent case–control study using the metagenomic shotgun sequencing technique, changes in gut microbiota were confirmed to be associated with the prevalence of Crohn's disease, and short-term exclusive enteral nutrition elicited limited impact on the overall composition of the microbiota in patients with Crohn's disease [26]. In the study, of the 49 patients with Crohn's disease, 35 patients (71%) had colonic involvements [26]. Fibre metabolised by intestinal bacteria and its absorption in the large bowel are required; therefore, fibre intake may work protectively on patients whose disease location is limited in the ileum (L1). Consequently, colonic involvement might cause the limited impact of exclusive enteral nutrition in the study and might also relate to the effectiveness of nutritional therapy to a newly diagnosed adult patient with Crohn's disease with ileal involvement [6]. Zinc plays an essential role in the function of the immune system and modulates the function of innate immune cells, including macrophages and neutrophils [19]. Zinc signals impact on NF-*κ*B activation; however, these findings have raised controversies [27]. Zinc deficiency induces intestinal membrane damage and inflammatory cell infiltration; conversely, zinc consumption maintains the membrane barrier integrity and prevents from massive neutrophil infiltration [28]. Vitamin D has effects on the regulation of the innate and adaptive immune systems, including Th1/Th17 T-cells and inflammatory cytokine decreases [29, 30].

In contrast, the role of fish and fatty acid consumptions still remains to be fully elucidated. A few studies, including a cohort study in the NHS II [18], reported the inverse correlation between fish intake and risk of Crohn's disease [31]. However, a Japanese case–control study indicated the consumption of fish was positively associated with Crohn's disease risk [32]. The effect of marine n-3 PUFAs on fish is a proposed potential mechanism to explain the inverse association between intake of fish and Crohn's diseases [18, 31]. Meanwhile, a clinical randomised controlled trial reported that treatment with n-3 PUFAs was not effective in the prevention of relapse in Crohn's disease [33]. A case–control study in children reported positive but nonsignificant associations between most fats and fatty acids and the risk of Crohn's disease. Moreover, they reported that n-3 PUFAs were negatively associated with Crohn's disease [34]. Interestingly, the other case–control study in children revealed diet–gene interactions between the dietary ratio of n-6 to n-3 PUFAs and single nucleotide polymorphisms (SNPs) in cytochrome P450 family 4 subfamily F member 3 (CYP4F3) or fatty acid desaturase 2 (FADS2), which are PUFA metabolic genes [35]. Specifically, the guanine and cytosine alleles of 2 SNPs, rs1290617 and rs1290620, in CYP4F3, are associated with high plasma levels of docosapentaenoic acid, which is one of the n-3 PUFAs. In the subjects who have those alleles, the ratio of n-6 to n-3 PUFAs was associated with higher odds ratio of Crohn's disease [35]. Equivalently, but except for SNP rs17831757, the high ratio of n-6 to n-3 PUFAs increased the odds ratio of Crohn's disease in the subjects who have the alleles of 3 SNPs in FADS2, which are associated with high plasma levels of n-3 PUFAs and low plasma levels of n-6 PUFA [35]. In contrast, the diet–gene interaction model has also been a controversial topic. A recent nested case–control study reported that no association was found between the ratio of n-6 to n-3 PUFAs and the risk of Crohn's disease under consideration of SNPs at CYP4F3 and FADS2 loci [36].

Oxidised n-3 PUFAs have anti-inflammatory effects, which may result in n-3 PUFAs acting as a ligand of peroxisome proliferator-activated receptor alpha (PPAR*α*) [37, 38]. This effect was observed with only oxidised n-3 PUFA, not unoxidised ones, in in vitro human umbilical vein endothelial cell experiments, which resulted from inhibitory effects on NF-*κ*B activation through a PPAR*α*-dependent pathway [38]. As mentioned above, zinc has inhibitory effects on NF-*κ*B activation. Indeed, a case–control study reported that zinc supplementation modulated docosahexaenoic acid levels in the red blood cell phospholipids [39]. Therefore, the dietary status of zinc may be a possible confounding factor in the relation between n-3 PUFAs and the development of Crohn's disease. Incidentally, an opposite result in mice experiments was reported [40]. In this report, consumption of oxidised n-3 PUFAs resulted in accumulation of 4-hydroxy-2-hexenal (4-HHE), an oxidised n-3 PUFA end product, in blood after its intestinal absorption and triggered oxidative stress and inflammation in the upper intestine. A previous study of 4-hydroxy-2-alkenals, 4-HHE, and 4-hydroxy-2-nonenal concentrations in a Korean foodstuff reported that the average daily exposure to 4-HHE was 1.6 *μ*g/day [41]. This report concluded that the value might not pose a significant risk for human health; however, excessive consumption of 4-HHE might increase the risk of Crohn's disease. No sufficient data is available concerning the safe level of 4-HHE; therefore, this may provide solutions to the problem regarding the controversial results in fish consumption.

Dietary fat is an important source of concentrated energy, together with other nutrients, and fatty acids are an important reservoir of stored energy [42]. They are stored as triacylglycerols in the body, which are principally from two sources, animal fats and vegetable oils [42]. The small intestine is a vital organ for triacylglycerol homeostasis; therefore, a better understanding of the mechanisms of intestinal fatty acid absorption is necessary to consider the role of fatty acids and meet consumption in Crohn's disease.

## 4. Malabsorption of Fatty Acids and Bile Acids in Crohn's Disease

Pancreatic lipase is essential in the digestion of dietary fats. Biliary bile acids form mixed micelles together with fatty acids, which function as a transport vehicle to deliver fatty acids to the apical membrane of enterocytes for absorption [42]. CD36 is one of the fatty acid transporters identified in the small intestine [42]. Despite in vitro experimental results with biopsy specimens from damaged and nondamaged colonic mucosa of 12 patients with inflammatory bowel disease, the number of CD36-positive cells was significantly lower in the damaged mucosa than in the nondamaged mucosa [43]. Even if n-3 PUFAs have anti-inflammatory effects, ineffective absorption of n-3 PUFAs can reduce the effects. Consequently, disease location and behaviour could have influenced on the dietary effect of fatty acids. This may be one possible reason why the controversial results in consumption of the various fatty acids, including n-3, were observed.

Bile acids are formed from cholesterol in the liver and secreted into bile [44]. Bile acids are concentrated in the gallbladder during the fasting state and secreted in the duodenum after stimulation by food [44]. Two primary bile acids, cholic acid and chenodeoxycholic acid, are synthesised in the liver, and gut microbiota produces secondary bile acids through two enzymatic reactions [45]. Most of the bile acids remain in the gut lumen until they reach the terminal ileum [44]. Bile acid uptake into the enterocyte occurs principally in the terminal ileum via the apical sodium-dependent bile acid transporter (ASBT) [46]. Reabsorbed bile acids enter hepatic portal circulation [46]. Bile acids are recycled with almost perfect yield (approximately 95%) [47], and their malabsorption can cause congenital diarrhoea, steatorrhoea, and reduced plasma cholesterol levels.

A recent case–control study suggested that bile acid malabsorption occurred in patients with Crohn's disease [46]. In the study, mRNA expression levels of ASBT, breast cancer-related protein (BCRP), sulfotransferase family 2A member 1 (SULT2A1), and fibroblast growth factor 19 (FGF-19) were significantly lower in inflamed regions in patients with active Crohn's ileitis than in controls. BCRP is a drug efflux transporter of the adenosine triphosphate-binding cassette (ABC) transporter family, which works in the opposite direction of a bile acid uptake transporter, ASBT. Meanwhile, SULT2A1 metabolises bile acids for protecting enterocytes from accumulation of bile acids in potentially harmful concentrations, and FGF-19 mediates the negative feedback regulation of hepatic bile acid synthesis between the gut and liver [46]. Therefore, bile acid transport and metabolism are reduced; increased bile acid concentrations appear associated with enhanced mucosal permeability and structural changes. Additionally, hepatic bile acid synthesis is enhanced due to missing FGF-19 signalling; intraluminal concentrations of bile acids may induce the onset of diarrhoea in Crohn's disease. As a result, bile acid malabsorption would lead to difficulty in revealing the positive effects of any kinds of foods by epidemiological studies.

The different aspect of bile acid malabsorption is seen in the relation to the CD4^+^ T effector (Teff) cell function in the ileum. The elevation of Teff cytokine expression in tissue is significantly associated with inflammatory bowel diseases [48, 49]. A recent study reported that multidrug resistance protein 1 (MDR1) expressing Teff plays a key role in mucosal homeostasis in the ileum [49]. MDR1 is one of the ABC transporter families. Therefore, MDR1 prevents bile acids from driving oxidative stress to intestinal T-cells [49], which may cause dysregulation of intestinal T-cells induced in Crohn's disease [1, 2]. Further studies paying more attention to bile acid handling in Crohn's disease not only in dietary dysmetabolism but also in immunological aspects are warranted.

## 5. Conclusions

The role of consumption of fatty acids in Crohn's disease remains to be discussed. This review provides an overview of the recent developments in the molecular pathways of lipid consumptions, which may connect immunological and nutritional studies in Crohn's disease, and epidemiological studies on disease location, phenotype, and diet. Data suggests that disease location, phenotype, and intestinal microbiota may be possible confounding factors. Additionally, bile acid may play a key role in the pathogenesis and/or exacerbation of Crohn's disease through bile acid malabsorption or dysregulation of negative MDR1-expressed Teff. Further comprehensive studies on diet and immunology may be warranted.
